# Understanding malaria vector dynamics and transmission in the Pouth-Kelle community, Nyong et Kelle division, Centre Region, Cameroon

**DOI:** 10.3389/fpubh.2026.1870575

**Published:** 2026-09-28

**Authors:** Elisabeth Melaine Ngo Bikoy, Philippe Bienvenu Nwane, Wolfgang Eyisap Ekoko, Serge Eteme Enama, Michael Junior Piameu Chadou, Leo Dilane Alenou, Stanislas Elysée Mandeng, Yannick Niamsi Emalio, Joseph Kamgno, Josiane Désirée Etang

**Affiliations:** 1 Laboratory of General Biology, University of Yaounde, Yaoundé, Cameroon; 2 Higher Institute of Scientific and Medical Research (ISM), Yaoundé, Cameroon; 3 Malaria Research Laboratory, Yaounde´ Research Institute (IRY), Organization for the Coordination of Endemic Diseases’ Control in Central Africa (OCEAC), Yaounde, Cameroon; 4 Yaoundé Teachers' Training College, University of Yaoundé, Yaoundé, Cameroon; 5 School of Health Sciences, Catholic University of Central Africa, Yaounde, Cameroon; 6 Department of Biological Sciences, Faculty of Medicine and Pharmaceutical Sciences, University of Douala, Douala, Cameroon; 7 Institute for Insect Biotechnology, Justus Liebig University Gießen, Gießen, Germany

**Keywords:** anopheline dynamics, Cameroon, malaria transmission, Pouth-Kelle community, vector behavior

## Abstract

**Introduction:**

Malaria remains a significant public health challenge in tropical regions. The disease is driven by multiple *Anopheles* vector species with varied ecological and behavioral traits. In transitional zones like the forested Pouth-Kelle community, detailed knowledge of vector dynamics and disease transmission remains limited. This study aimed to characterize local *Anopheles* species composition, biting behavior, and malaria transmission indicators to guide control strategies.

**Materials and methods:**

From February 2021 to January 2022, adult mosquitoes were monthly collected indoors and outdoors over two consecutive nights in three dwellings using “*human landing catch*” method. Morphological and molecular techniques were used for species identification. Entomological indicators were assessed, and an ELISA assay was conducted to detect *Plasmodium falciparum* circumsporozoite proteins in order to determine infection rate. Concurrently, residents were screened for malaria by microscopy. Statistical analysis examined the relationship between infection rate, age, and sex.

**Results:**

A total of 790 *Anopheles* mosquitoes were collected. Of these, *An. gambiae* s.l. accounted for 64.7% of the sample, with *An. gambiae* comprising 98% and *An. coluzzii* 2% of that group. Other species included *An. nili* (20%), *An. funestus* (15.2%), and *An. paludis* (0.1%). *An. gambiae* s.l. biting rates were 6.86 bites/person/night outdoors and 6.66 bites/person/night indoors; *An. funestus* had 1.47 bites/person/night outdoors and 1.67 bites/person/night indoors; *An. nili* registered 2.32 bites/person/night outdoors and 2.00 bites/person/night indoors. Infection rates for *An. gambiae* s.l. differed significantly by location, with 11.3% indoors compared to 6.48% outdoors (*p* = 0.039), whereas infection rates for *An. funestus* and *An. nili* were similar indoors and outdoors. Entomological inoculation rates (EIR) were consistently higher indoors for all species, particularly for *An. gambiae* s.l. (0.75 infective bites/person/night indoors vs. 0.45 infective bites/person/night outdoors). Malaria prevalence among humans was 30.8%, with no significant difference between sexes; however, a marked decline in prevalence was observed with increasing age. Children under 10 years had 28.5 times higher odds of infection compared to adults aged 30–40.

**Conclusion:**

Multiple *Anopheles* species sustain malaria transmission in Pouth-Kelle, exhibiting diverse biting behaviors and varying infection rates both indoors and outdoors. Elevated indoor infection rates and EIR for *An. gambiae* s.l support maintaining indoor control interventions, while outdoor transmission calls for integrating strategies targeting both environments, prioritizing vulnerable groups, especially children.

## 1 Background and Rationale

Mosquito-borne diseases remain a serious health concern in tropical and subtropical areas of the world (1). Among these diseases, malaria is the leading cause of illness and death, with an estimated 282 million cases and approximately 610,000 deaths reported globally in 2024, showing a slight increase compared to previous years (2, 3). Former studies have shown that ≈ 58% of malaria deaths and a significant proportion of cases are found in the world’s poorest countries, with the highest incidence rates occurring among the poorest 20% of the population (4). Similar to other parasitic diseases, the sub-Saharan African region bears the heaviest burden of malaria, accounting for 94% of all cases and 95% of all deaths. Children under 5 years of age are the most vulnerable group, representing approximately 80% of all deaths related to the disease in Africa (2).

In Cameroon, malaria represents a critical public health challenge, putting over 90% of the population at risk of infection (5). Each year, the country reports ≈ 6 million of cases and 11,000 deaths, with more than 70% of the population living in areas characterized by intense, year-round transmission (6). However, the intensity of disease transmission is not uniform and varies widely across ecological zones. Transmission is typically higher in rural, forested areas compared to drier and urban environments (7). The disease divides the country into three main epidemiological facies, based on the duration of transmission: (1) the Sahelian facies lasting 3 to 4 months annually; (2) the tropical facies occurring for 6 to 9 months; and (3) the equatorial facies, characterized by continuous year-round transmission (8). Despite this classification, malaria transmission remains highly heterogeneous within each facies. Studies have documented significant variations in transmission intensity between neighboring villages, as well as between rural and urban settings within the same region (9, 10). This heterogeneity is influenced primarily by abiotic factors such as temperature, rainfall, and humidity, which shape the seasonal and geographic patterns of malaria transmission. Moreover, local factors including human land use, urbanization, and socio-economic conditions also significantly affect the intensity, seasonality, and distribution of malaria in Cameroon (11–13).

Combined environmental factors shape the habitats and ecological preferences of malaria vector species, with some species exhibiting distinct preferences within endemic regions (14–16). Species such as *An. arabiensis* and *An. rufipes* are known to colonize Sahelian and savannah areas (17–19), while *An. moucheti* is known as a forest species (20–22), *An*. *melas* and *An. merus* are African coastal mosquito species that inhabit and breed in saltwater environments (23–25). Increasingly, species such as *An. gambiae*, *An. coluzzii* and An. *stephensi* are revealing a great adaptive plasticity to a wide range of ecological environments (26–28), exhibiting distinct feeding preferences: some primarily feed on humans, others on animals, and some on both. Additionally, certain species bite indoors, others outdoors, and some in both environments, demonstrating highly developed adaptive abilities (29, 30).

This behavioral diversity, especially vectors’ adaptation in biting locations and times (indoors vs. outdoors, nocturnal vs. crepuscular), presents a major challenge to the effectiveness of current vector control tools, such as long-lasting insecticidal nets (LLINs) and indoor residual spraying (IRS). These interventions primarily target endophagic (indoor-biting) and endophilic (indoor-resting) mosquito species and allow exophagic and exophilic vectors to evade (15). Numerous studies have reported that certain vectors modify their biting behavior by shifting their activity to dawn or dusk or increasing outdoor biting, thus avoiding contact with LLINs and IRS (31, 32). Such behavioral plasticity can significantly reduce the impact of conventional interventions on malaria transmission, particularly in regions where *An. arabiensis* or *An. funestus* are predominant (33). Therefore, before deploying these tools, it is essential to conduct a comprehensive assessment of local vector behaviors or to update existing entomological data, in order to optimally tailor control strategies, including complementary measures targeting exophagic and exophilic species (34). In fine, a thorough understanding of the malaria vector system, both at small and large scales appears as a crucial prerequisite for guiding the use of control tools (35, 36). Unfortunately, the majority data on malaria vectors and transmission primarily come from agro-industrial zones, major cities, and large urban development projects, including infrastructure such as roads and dams (37–39).

However, certain strategic areas that are likely to sustain the transmission of vector-borne diseases remain largely neglected and underexplored (40, 41). These zones can serve as important reservoirs for the resurgence and outbreaks of epidemic cases in major urban centers, where surveillance is generally more intensive. The Nyong and Kelle Division, a transitional area between the cities of Douala and Yaoundé, is one example among many in Cameroon that remains largely unexplored, or very poorly studied, with regard to the bionomics of disease vectors and pathogen transmission. Apart from the malaria cases diagnosed in local health facilities within this vast geographic area, no epidemiological or entomological studies on malaria transmission have, yet been conducted in this area.

The Pouth Kelle community was selected as a sentinel site to conduct comprehensive investigations of malaria dynamics in the area. Therefore, assessing epidemiological and entomological indicators is essential for understanding malaria vector transmission locally and, to a lesser extent, at the zonal level, to provide information on the implementation of appropriate control measures and optimize existing interventions. This study examines malaria vector behaviors and transmission patterns in the Pouth Kelle community, Eseka Health District, Center Region of Cameroon.

## 2 Materials and methods

### 2.1 Study site and period of the study

The study was conducted from February 2021 to January 2022 in the Pouth-Kelle community (3°84,667 N; 10°812061E; 232 m), located in the forest area of Eseka health District, in the Nyong and Kelle Division, Center Region of Cameroon (Figure 1). The community is situated along the Boumyebel-Eseka road, approximately 10 km from Boumyebel, which lies on National Highway N°3 connecting Douala and Yaoundé cities, the economic and political capitals of Cameroon, respectively. This location falls within the Guinean equatorial zone, characterized by four distinct seasons: two dry seasons (December–February and July–August) and two rainy seasons (March–June and September–November). The mean annual temperature ranges between 23 °C and 25.5 °C, while average annual rainfall varies from 1,500 mm to 1,800 mm. The vegetation cover consists primarily of Guinean-Congolian semi-deciduous rainforest, which has been influenced by human activities that promote the establishment of palm groves, cocoa plantations, and food crops such as plantain, banana, and cassava. The local hydrographic network is dominated by the Nyong River and its main tributaries, namely La Kelle, Maloume, Mouanda, and Djogob. Overall, the climate exhibits a cyclical pattern of dry and rainy seasons, creating favorable conditions for insect development and ongoing mosquito-borne disease transmission.

**Figure 1:**
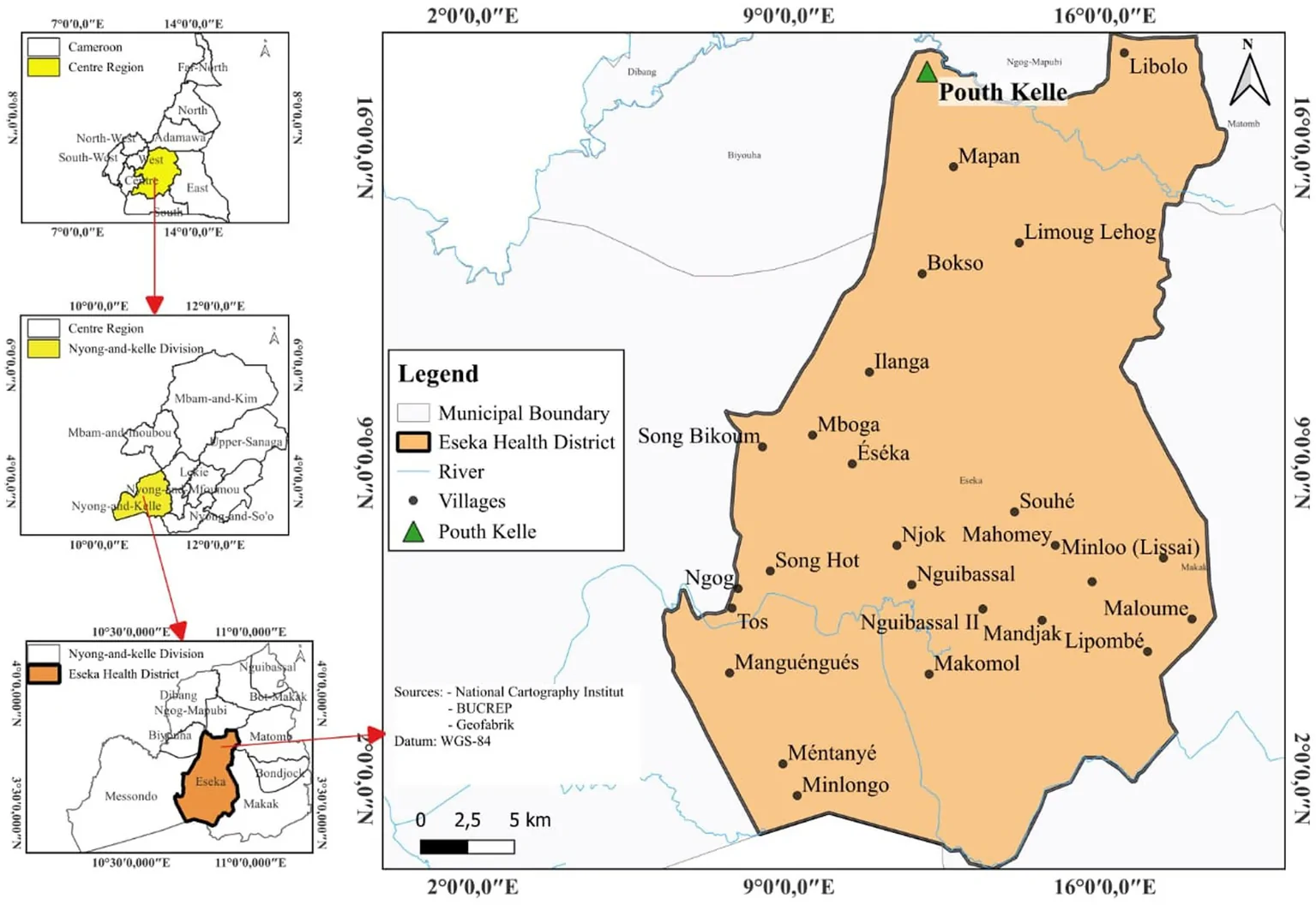
Map of Cameroon showing the Nyong et Kelle division and the location of Pouth Kelle community.

### 2.2 Ethics approval and consent to participate

The study was conducted under the ethical clearance *No 2,263/CRERSSH/2021* delivered by the Cameroon Regional Ethics Committee for Research on Human Health (CRERSH). The purpose of the study was to collect entomological and epidemiological indicators of malaria transmission with the approval of the community leader and his representatives. The community members were informed that participation in the study was voluntary with no sanctions for withdrawal. All adult men or women who took part in the data collection signed a written informed consent form before being enrolled in the study as recommended by the validated study protocol. At the first suspicion of malaria infection, and confirmation by rapid diagnostic test, volunteers were provided with the curative treatment recommended by the National Malaria Control Program in Cameroon.

### 2.3 Entomological survey

- Field sampling of adult mosquitoes

Adult mosquitoes were collected over a 12-month period, from February 2021 to January 2022, using the *human landing catch* (HLC) technique as described by the WHO (42). Collections were carried out by local trained volunteers aged from 25 to 50 years. The collections were performed during two consecutive nights each month, from 6:00 p.m. to 6:00 a.m., in three selected dwellings, each situated in one of the three blocks comprising the community. These three dwellings were selected to provide a balanced geographic representation of the study site, in order to avoid biases related to the position of collection points. In each dwelling, mosquitoes were collected both indoors and outdoors by two teams, each consisting of two volunteers. The first team worked from 6:00 p.m. to midnight, and the second from midnight to 6:00 a.m. During each collection period, the two team members rotated every 2 h between indoors and outdoors. Mosquitoes collected during each 2-h interval were placed in a textile bag labeled with the dwelling number, collection environment, and time slot. Female adult mosquitoes were then sorted by genus based on prominent morphological traits, with anophelines identified morphologically using the identification keys of Gillies & de Meillon (43) and Gillies & Coetzee (44). Samples of anophelines were individually stored in 1.5 mL Eppendorf tubes containing silica gel and labeled with the mosquito’s identification number. An Excel spreadsheet was created to record, for each mosquito, its identification number, time of collection, collection environment, dwelling number, and full date (day, month, and year). All Eppendorf tubes were stored in boxes at −20 °C for subsequent laboratory analyses.

- Laboratory analysis of *Anopheles* mosquitoes

All female mosquitoes morphologically identified as members of the *Anopheles gambiae* complex were subjected to molecular identification. DNA was extracted from legs and wings using 2% CTAB (cetyltrimethylammonium bromide), following the protocol described by Collins and colleagues (45). Polymerase chain reaction (PCR) amplification was then performed to determine the sibling species within the *Anopheles gambiae* complex, according to the method of Fanello and colleagues (46). The primers included (1) UN: [5′-GTG TGC CCC TTC CTC GAT GT-3′], (2) GA: [5’-CTG GTT TGG TCG GCA CGT TT-3′], (3) AR: [5′-AAG TGT CCT TCT CCA TCC TA-3′]; (4) ME: [5′-TGA CCA ACC CAC TCCCTT GA-3′] and PCR amplifications were performed using an AB Bioapplied Systems 2,720 thermocycler, serial number 4359659, manufactured on April 1, 2011. Additionally, the ELISA technique was applied to the head and thorax of each anopheline specimen to detect the *Plasmodium falciparum* circumsporozoite protein (CSP), using the procedure established by Burkot and colleagues (47) and modified by Wirtz et and colleagues (48).

### 2.4 Parasitological survey for malaria diagnosis

- Selection of participants

The study was conducted in a community of ≈ 300 inhabitants. The sample size was determined using formula as described by Charan and Biswas (49) as follows.

$n=\frac{Z_{a/2}^{2}\times p(1-p)}{d^{2}}$

With n as the number of samples, p an estimated malaria prevalence of 50% (*p* = 0.5), at a 95% confidence interval (Zα/2 = 1.96) and a 5% margin of error (d = 0.05), the calculated sample size was 384 participants.

The study participants were native residents who had lived in the community for at least 3 months. Written informed consents were obtained from all participants prior to enrolment, without offering any incentives. For minors aged 5 to 17 years, consents were obtained from parents or legal guardians. Individuals presenting with febrile illnesses or those who had received antimalarial treatment within the previous 2 weeks were excluded from the study. Data collection involved two steps: (1) registration and recording of socio-demographic information, including name, age, sex, occupation, place of residence, and use of preventive measures; and (2) collection of blood samples. Each participant was assigned a unique identification code to ensure confidentiality and facilitate data analysis (50).

- Blood sampling, preparation and staining of smears

Finger-prick blood samples were obtained from each participant using sterile, single-use lancets. The first drop of blood was wiped away with a dry cotton wool. Thick and thin blood films were prepared following the method described by Cheesbrough (51). Each slide was labeled with the participant’s unique identification code, and the blood films were air-dried while being protected from dust and flies. In the field laboratory, thin films were fixed in 100% methanol for 1 min, after which both thick and thin smears were stained with 3% Giemsa solution for 30 min (51). Slides were examined under a light microscope using the oil immersion objective lens at ×100 magnification. The bench aid for malaria parasite diagnosis was employed to identify and confirm the presence of malaria parasites (52). Species identification was performed on the thin films, while parasite density was determined using the thick films. Parasite trophozoites were counted against 200 white blood cells (WBCs), assuming a total WBC count of 8,000 cells/μL of blood (53). A smear was deemed negative if no parasite was detected after examining 100 high-power fields.

### 2.5 Data analysis

Man biting rate (m.a) which refers to the average number of bites per person per night (bites/person/night) for the vector species (also called the aggressiveness of the species) was calculated as the total number of *Anopheles* mosquitoes collected divided by the number of collectors. Infection rate (IR) was calculated as the proportion of *Anopheles* species tested positive for *P. falciparum* CSP by ELISA. The Entomological Inoculation Rate (EIR) was determined as the product of the Infection Rate (IR) and the man biting rate (m.a). The endophagy index was defined as the ratio of the number of mosquitoes collected indoors divided by the total number of mosquitoes collected both indoors and outdoors. Data were analyzed using SPSS Statistics 17.0. A Chi-square test was used to assess the significance of variables, with a threshold set at *p* < 0.05. For *Plasmodium* infection data in humans, descriptive analyses of categorical variables were reported as absolute and relative frequencies. Parasite densities were classified as mild (<1,000 parasites/μL), moderate (1,000–4,999 parasites/μL), or severe (≥5,000 parasites/μL) according to WHO (53) and Cheesbrough (51). Associations between variables (sex, age) and malaria infection were evaluated using bi-variate analysis with the Chi-square test. A multivariate binary logistic regression model was constructed with malaria infection status (infected = 1, not infected = 0) as the dependent variable. Independent variables included sex (male = 0, female = 1) and age (categorized into [age groups] or treated as a continuous variable). Results are presented as odds ratios (OR) with 95% confidence intervals, and statistical significance was set at *p* < 0.05. All statistical analyses were conducted using R software (version 4.5.0), with significance set at 5%.

## 3 Results

### 3.1 Entomological survey

- Composition and abundance of Anopheline mosquitoes

A total of 790 *Anopheles* mosquitoes were collected throughout the study period. Morphological identification revealed four species: *Anopheles gambiae* s.l (64.68%; *N* = 511), *An. nili* (20%; *N* = 158), *An. funestus* (15.2%; *N* = 120), and *An. paludis* (0.1%, *N* = 1; Figure 2). Molecular analyses were conducted on a randomly selected subset comprising 30% of the *An. gambiae* s.l. specimens (*N* = 159) from the monthly collections. The results indicated the coexistence of two sibling species within the *An. gambiae* complex: *An. gambiae*, which was the predominant species (≈ 98%; *N* = 156), and *An. coluzzii*, representing a minority (≈ 2%; *N* = 3).

- Mosquito aggressiveness indoors and outdoors and entomological indicators of malaria transmission

**Figure 2:**
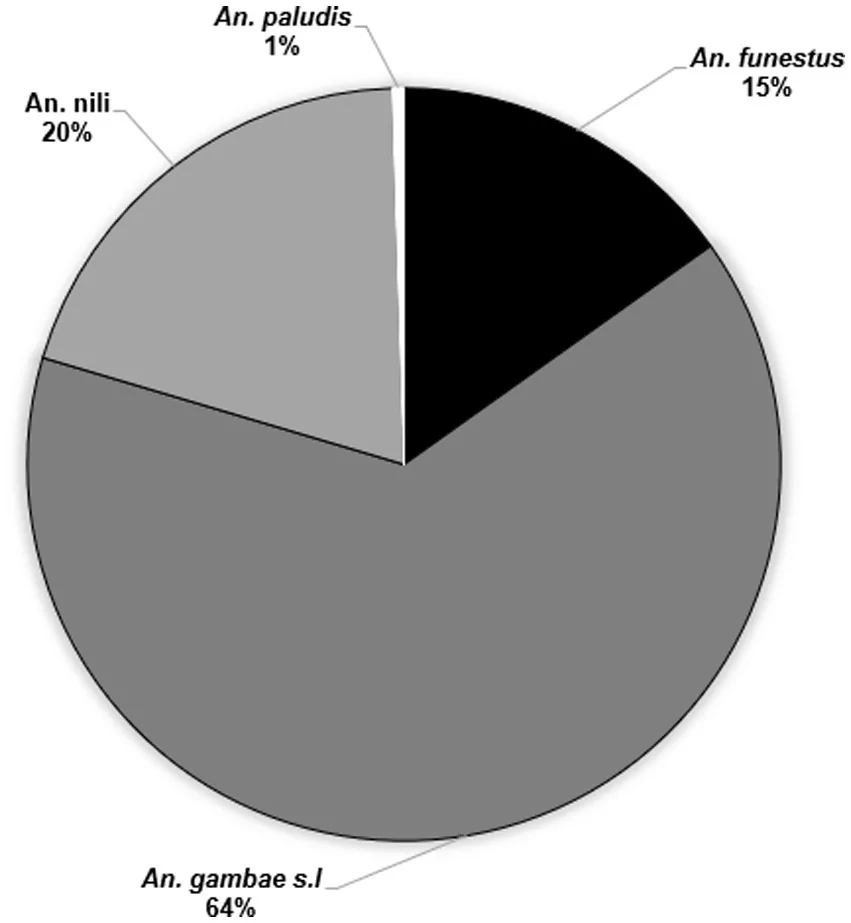
Composition of the Anopheline fauna sampled during the study period.

The monthly and hourly biting activities of the collected *Anopheles* species were analyzed throughout the study period. Figure 3 displays the biting activity profiles of the three main species identified at the study site including An. *funestus* (3A), *An. nili* (3B), and *An. gambiae s.l*. (3C). Overall, these mosquito species exhibited similar biting patterns both indoors and outdoors, with peak activity occurring during short rainy season from March to April. During this period, *An. nili* showed the highest peak in biting activity, followed by *An. funestus*, and then *An. gambiae* s.l. Between the end of the short rainy season (March–June) and the beginning of the dry season (July–August). *An. gambiae* s.l. became the predominant species, while *An. funestus* and *An. nili* were minor species. From August to January, period corresponding to the long rainy and dry seasons, the densities of all the three species declined sharply, each persisting only as a residual population.

**Figure 3:**
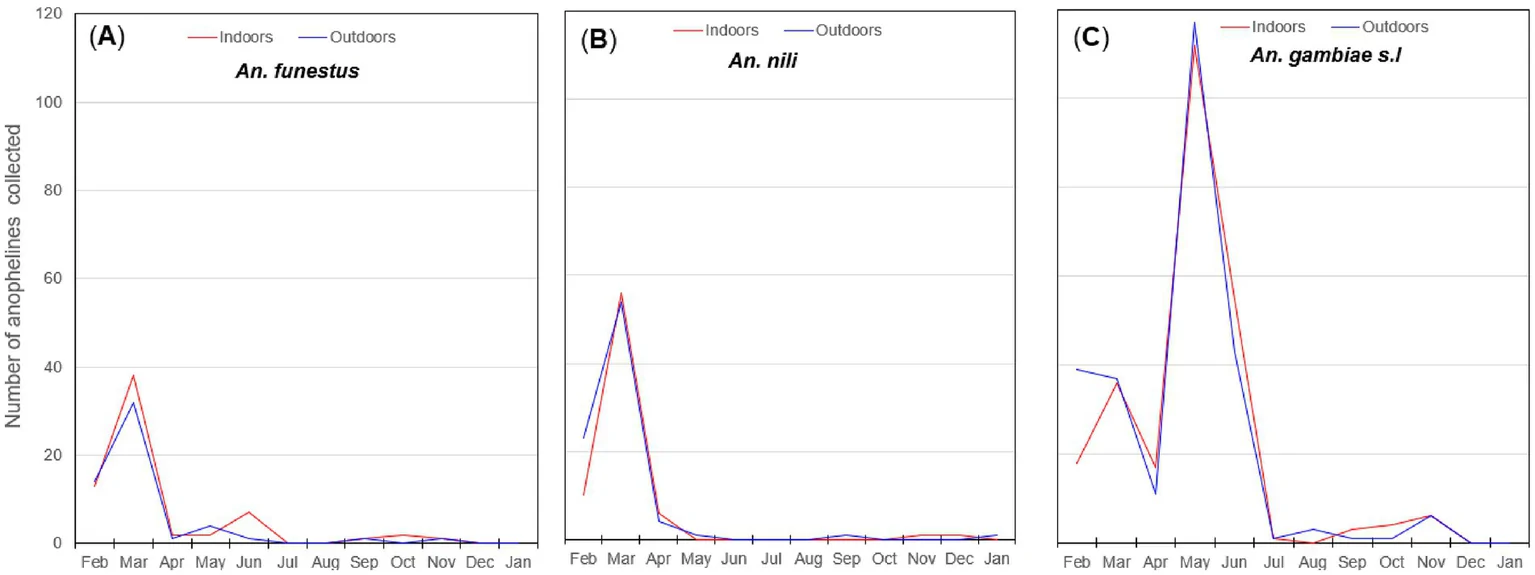
Monthly biting activity of malaria vectors collected indoors and outdoors. **(A)** An. funestus, **(B)** An. nili, and **(C)** An. gambiae s.l.

Regarding the hourly biting activity shown in Figure 4, the three species exhibited distinct aggressiveness profiles both indoors and outdoors. At both indoor and outdoor locations, each species remained active throughout the collection period from 6:00 p.m. to 6:00 a.m., with peak biting times differing between species. *An. gambiae* and *An. funestus* exhibited significant biting activity between 10:00 p.m. and 4:00 a.m., with a peak indoors between midnight and 2:00 a.m., and a peak outdoors between 2:00 a.m. and 4:00 a.m. In contrast, *An. nili* was active between 8:00 p.m. and midnight, showing a peak indoors from 10:00 p.m. to midnight, and outdoors between 6:00 p.m. and midnight, with the highest activity occurring between 8:00 p.m. and 10:00 p.m.

- Entomological indicators of malaria transmission

**Figure 4:**
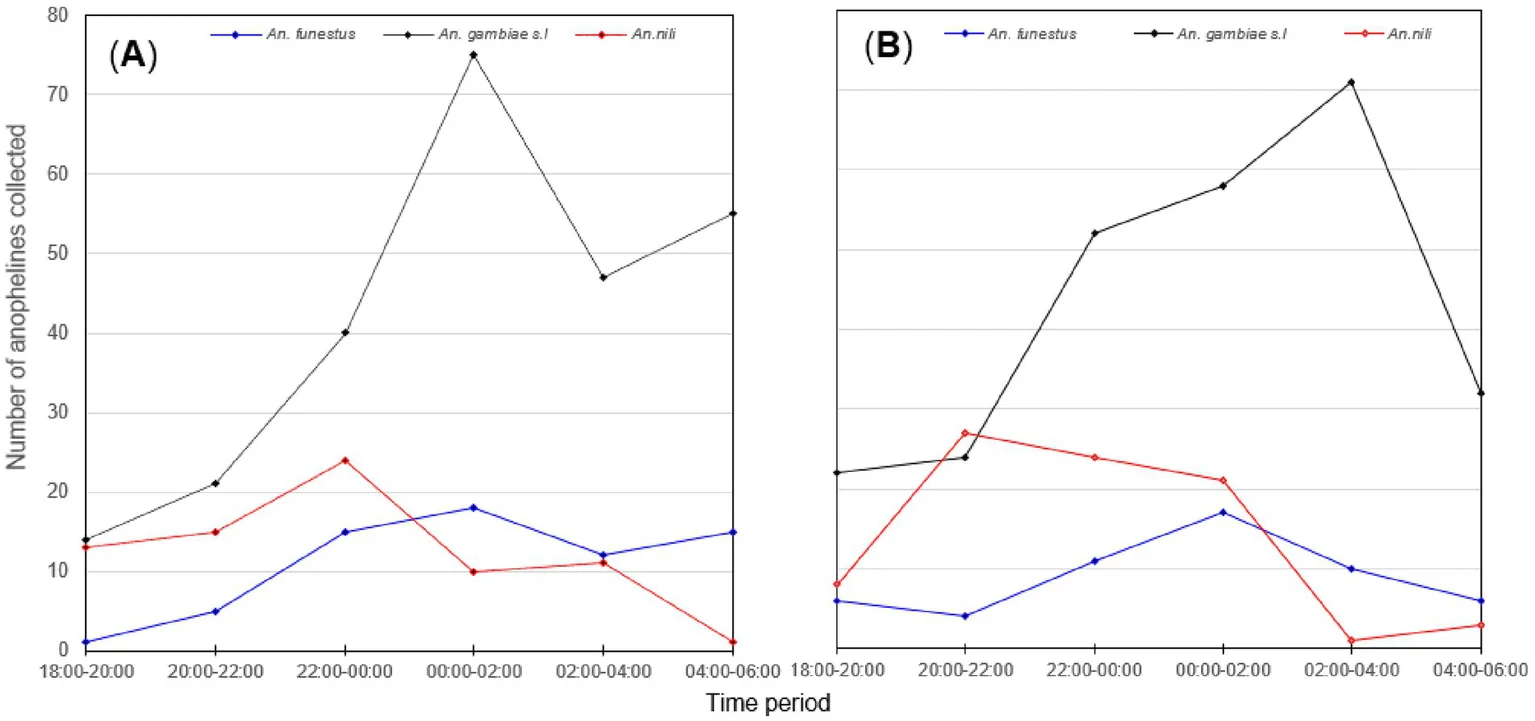
Hourly biting activity of anopheles mosquitoes indoors and outdoors. **(A)** Indoors; **(B)** outdoors.

The entomological indicators of malaria transmission assessed in this study are from a total of 755 mosquitoes collected over 6 months, from February to July, with 373 collected indoors and 382 outdoors. These two subsamples consisted of *An. funestus*, *An. gambiae s.l*, and *An. nili* at respective proportions of 53.6, 49.2, and 46.8% indoors, and 46.1, 50.8, and 50.6% outdoors (Table 1). Across all mosquito species collected, the biting rates ranged from 1.47 bites per person per night (b/p/n) for *An. funestus* outdoors to 6.86 b/p/n for *An. gambiae* s.l. outdoors, depending on the species and the sampling location. Overall, *An. gambiae* s.l. showed relatively higher biting rates both indoors and outdoors compared to *An. funestus* and *An. nili*, which displayed similar biting rates in both locations (Table 1). Whether considering each species individually or the combination of collected species, the biting rate indoors was comparable to that outdoors (*p* > 0.05). The overall endophagy rate recorded was approximately 50%, with species-specific rates of 53.6% for *An. funestus*, 48.9% for *An. gambiae* s.l., and 46.7% for *An. nili* (Table 1).

**Table 1:** Aggressiveness of anopheline species indoors and outdoors, endophagy, and malaria transmission indicators.

Mosquito species	Indoors					Outdoors						Indoor-outdoor comparison (*p*-value)		
	N (%)	Biting rate [95%CI]	Spz^+^	Infection rate [95%CI]	EIR	N (%)	Biting rate [95%CI]	Spz^+^	Infection rate [95%CI]	EIR	EI	Biting rate	Infection rate	EIR
*An. funestus*	62 (53.9)	1.72 [1.33–2.21]	5	8.06 [2.70–17.58]	0.14 [0.04–0.32]	53 (46.1)	1.47 [1.08–1.93]	4	7.55 [2.09–18.47]	0.11 [0.03–0.28]	53.6	0.27	0.99	0.4
*An. gambiae s.l*	239 (49.2)	6.64 [5.84–7.52]	27	11.30 [7.63–16.20]	0.75 [0.51–1.06]	247 (50.8)	6.86 [6.04–7.76]	16	6.48 [3.92–10.33]	0.44 [0.26–0.72]	48.9	0.80	**0.03**	0.06
*An. nili*	72 (46.8)	2.00 [1.570–2.53]	1	1.39 [0.04–7.23]	0.03 [0.00–0.15]	82 (52.2)	2.28 [1.82–2.83]	1	1.22 [0.03–6.75]	0.03 [0.00–0.15]	46.7	0.45	1	1
Total	373 (49.4)	10.36 [9.36–11.42]	33	8.85 [6.26–12.29]	0.92 [0.63–1.30]	382 (50.6)	10.61 [9.58–11.74]	21	5.50 [3.43–8.53]	0.58 [0.36–0.90]	49.5	0.73	0.13	0.12

N, Number tested; CI, confidence interval; Spz^+^, number positive for sporozoite; EIR, Entomological inoculation rate; EI, Endophagy index.

The bold values indicate statistically significant results (*p* < 0.05).

The infection rates ranged from 1.22% for *An. nili* outdoors to 11.30% for *An. gambiae* s.l indoors. Overall, infection rates were 8.85% indoors and 5.50% outdoors, showing no significant difference between the two locations (χ^2^ = 3.68; df = 1; *p* = 0.055; Table 1). This general trend in infection rates observed when all species are combined is also seen in *An. funestus* and *An. nili*, with indoor rates slightly higher but not significantly different from outdoor rates. In contrast, *An. gambiae* demonstrated a significantly higher infection rate indoors at 11.30% compared to 6.48% outdoors (χ^2^ = 4.22; df = 1; *p* = 0.039). The entomological inoculation rates (EIRs) ranged from 0.03 ib./p/n, both indoors and outdoors for *An. nili*, to 0.75 ib./p/n indoors for *An. gambiae*. Whether considering species individually or combined, the recorded inoculation rates were slightly higher indoors than outdoors, with however no significant difference (*p* > 0.05), except for *An. nili*, where the rates were identical indoors and outdoors (Table 1).

### 3.2 Parasitological survey

- Socio-demographic data

A total of 107 participants were recruited in the surveyed community for parasitological study, including 54.2% males and 45.8% females. The median age was 43 with a minimum of 5 years and a maximum of 85 years. Table 2 summarizes the socio-demographic characteristics of the study population. The most represented age groups were [30–40] years and [60–70] years, accounting for ≈ 20 and 18% of the study population, respectively. The overall prevalence of malaria infection was 30.8% (95%CI: 22.1–39.6%).

- Distribution of malaria infection within the population.

**Table 2:** Descriptive, univariate, and logistic regression analyses of malaria infection by sex and age.

Variable	N (%)	Tpz^+^	Prevalence [95%IC] (%)	OR [95%IC]	*p*-valeur OR
Sex					
Male	58 (54.2)	17 (15.9)	29.3[17.6–41.0]	0.415 -Reference	-
Female	49 (45.8)	16 (14.9)	32.7[19.6–45.7]	0.485 [0.514–2.657]	-
Age group					
<10	7(6.5)	5(15.1)	71.4 [35.9–93.7]	28.5 [4.8–200]	**< 0.001**
[10–20]	5(4.8)	3(9.1)	60.0 [17.0–92.7]	14.8 [2.2–110]	**0.005**
[20–30]	15(14.0)	7(21.2)	46.7 [21.3–73.9]	8.1[1.7–49]	**0.010**
[30–40]	21(19.6)	2(6.1)	9.5 [2.6–31.0]	Reference	-
[40–50]	14(13.1)	3(9.1)	21.4 [7.4–47.7]	2.7 [0.5–15]	0.240
[50–60]	15(14.0)	4(12.1)	26.7 [10.1–54.6]	3.3 [0.6–18]	0.180
[60–70]	19(17.7)	7(21.2)	36.8 [17.2–61.7]	5.8 [1.4–30]	0.020
≥ 70	11(10.3)	2(6.1)	18.2 [4.5–47.7]	2.0 [0.3–10]	0.550
Total	**107**	**33**	**30.8 [22.1–39.6]**	**3.4 [1.2–9.5]**	**0.011**

Tpz^+^, Number positive for trophozoites; N, number; OR, Odd ratio; IC, confidence interval.

The bold values indicate statistically significant results (*p* < 0.05).

The distribution of malaria infection within the studied population is presented in table 2. The *p*-values of 0.415 for males and 0.485 for females, coupled with odds ratios ≈ 1, indicate no statistically significant association between sex and malaria infection risk, suggesting similar prevalence between males and females in the community (Table 2). A significant association between age and parasite infection was confirmed by the Chi-square test (χ^2^ = 24.5; df = 7; *p* < 0.001), with higher prevalence observed in younger individuals compared to older age groups. The logistic regression further highlights this trend, showing that the youngest groups (i.e., < 10, 10–20, and 20–30 years) face a significantly increased risk of infection, particularly children under 10 years old (OR = 28.5). This risk steadily decreases with age, reaching its lowest level in the 30–40-year group, which serves as the reference group. Beyond this reference group, the older age groups namely [40–50] (OR = 2.7), 50–60 (OR = 3.3), and 70 years and above (OR = 2.0) did not show a statistically significant difference in infection risk compared to the reference group (*p* > 0.05).

However, the [60–70] year group exhibited a moderately significant increase in risk (OR = 5.8; *p* < 0.05). Out of the 33 individuals diagnosed positive for malaria (30.8%), 2 (≈ 6%) carried gametocytes, while 31 (≈94%) harbored trophozoites, indicating an early stage of infection. Trophozoite density ranged from 32 to 4,000 per microliter of blood. Among the 31 cases with trophozoites, 80.6% (*n* = 25) were classified as having mild infections, and 19.4% (*n* = 6) had moderate infections, no severe infections were reported (Figure 5).

**Figure 5:**
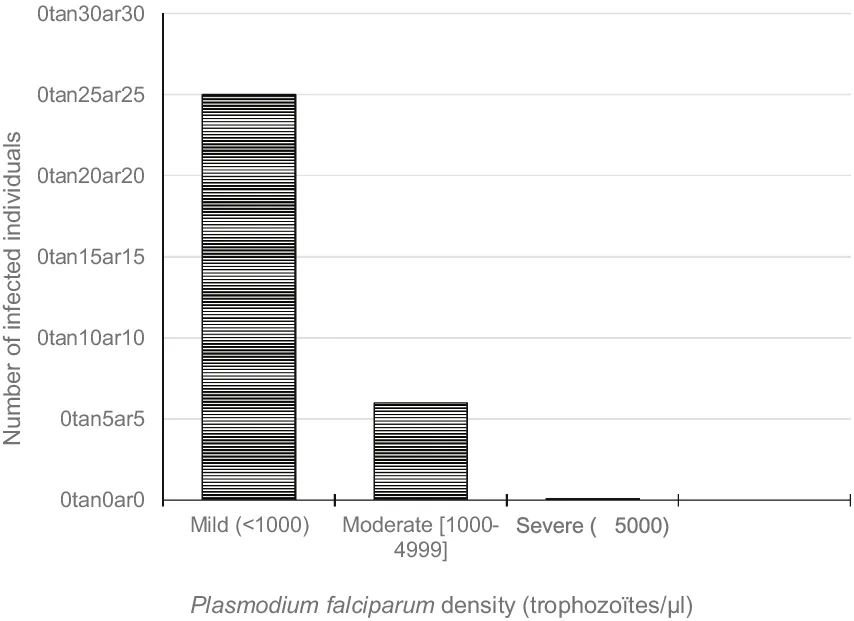
Classification of malaria-infected individuals in the Pouth-Kelle community based on trophozoite density in blood.

## 4 Discussion

This study provides in-depth assessment of the entomological and parasitological characteristics of malaria transmission in the Pouth-Kelle community. The diversity of anopheline species observed including *An. gambiae*, *An. coluzzii*, *An. funestus*, *An. nili*, and *An. paludis* reflects the heterogeneous ecological environments of the area, typical of many sub-Saharan regions where members of the *Anopheles gambiae* complex (s.l.) coexist with other anopheline species within overlapping geographic zones (44, 53, 54). The predominance of *An. gambiae* among the *An. gambiae* complex samples aligns with its ability to adapt and thrive across diverse habitats, including rural and peri-urban areas throughout sub-Saharan Africa (43, 55, 56). The occurrence of species such as *An. nili* and *An. funestus* reflects specific local ecological conditions, with *An. nili* particularly breeding in fast-flowing, well-oxygenated streams and rivers commonly found in forested or riverine environments, like the La Kelle River (57, 58); and *An. funestus* utilizing more permanent aquatic habitats with emergent vegetation (43, 53). Although less represented, *An. paludis* could play an important role in transmission, especially as ecological changes might increase its epidemiological relevance (58). The coexistence of all these mosquito species underlines a diverse mosaic of ecological niches with minimal competition, collectively supporting the potential for year-round malaria transmission (59, 60). Such complexity underscores the value of strengthened entomological surveillance that accounts for seasonal dynamics and species interactions to optimize vector control (61, 62).

The highest densities of all identified species in our study site were recorded during the short dry and rainy seasons, which are generally favorable for mosquito development and proliferation. In contrast, during the long dry and rainy seasons, vector populations were almost absent. These results suggest that rainfall patterns have a significant influence on the abundance and biting activity of mosquito populations at the study site (63). Indeed, rainfall creates larval habitats which increases mosquito proliferation (64), this observation is consistent with the seasonal densities of the *Anopheles* species reported in this study (65).

In the context of this study, species dominance shifts temporally with *An*. *nili* and *An. funestus* predominating in the short rainy season, while *An. gambiae s.l.* dominates from April to July, likely reflecting species-specific breeding preferences and adaptability to environmental changes (66, 67). Conversely, vector populations sharply decline during the long rainy and dry seasons (August to January), corresponding to unfavorable conditions that suppress development, with mosquitoes surviving in residual populations sustaining low-level malaria transmission (68).

Differences in species-specific biting times reflect ecological adaptations impacting exposure risks. *An. nili* exhibits crepuscular and early nocturnal outdoor biting (18:00–00:00), increasing early evening transmission risk and potentially limiting the protective efficacy of indoor interventions such as long-lasting insecticide nets (LLINs) and indoor residual spraying (IRS) (69–72). In contrast, *An. gambiae s.l.* and *An. funestus* tend to bite later at night to early morning, coinciding with human indoor resting periods and thus remaining susceptible to these indoor-focused control measures (73). Indoor biting rates of *An. gambiae s.l.* in this study confirm the importance of indoor transmission routes (74, 75). However, the similar indoor and outdoor biting and infection rates recorded for *An. funestus* and *An. nili*, along with endophagy rates close to 50%, reveal an opportunistic or amphiphilic feeding behavior, indicating that these mosquito species exploit both environments (57, 76, 77). These findings emphasize that malaria transmission occurs not only indoors but also outdoors (78–80), highlighting the importance of integrating both indoor and outdoor control strategies to ensure effective disease surveillance (81–83). Data recorded particularly entomological inoculation rates reinforce these transmission patterns. *An. gambiae s.l.* exhibited significantly higher EIR values indoors compared to outdoors, emphasizing its indoor transmission efficiency and supporting continued reliance on LLINs and IRS (84, 85). In contrast, *An. funestus* and *An. nili* showed no significant indoor-outdoor difference, suggesting that indoor interventions alone may be insufficient to fully control the transmission potential (86, 87). Collectively, this vector behavioral heterogeneity complicates control efforts and advocates for a multifaceted approach tailored to local vector ecology.

The biting rates and Entomological Inoculation Rates (EIRs) reported in this study varied depending on the *Anopheles* species collected and the environment (indoor versus outdoor).

The varying *Plasmodium* infection rates among the reported *Anopheles* species, as well as the respective biting and entomological inoculation rates (EIRs), suggest active malaria transmission in the Pouth Kelle community. However, the values of these indicators are relatively low compared to those reported in previous studies conducted in other locations of the Center region. Specifically, the human biting rates observed here are markedly lower than the rates of 21.45 and 45.5 reported in Makenene and Bakeng (88, 89). These disparities may result from variations in local vector species composition, seasonal dynamics, environmental conditions, and human behaviors affecting exposure. The reported global EIR values are also significantly lower than the 3.8 EIR reported in Bankeng (89), indicating a lower intensity of malaria transmission in the Pouth Kelle community. These findings underscore the heterogeneous nature of malaria transmission in the Center region (90), reflecting both temporal and spatial variation in mosquito abundance and the epidemiological diversity across different locations. In fine, the entomomogical data reported in Pouth Kelle are consistent with seasonal transmission influenced by climatic variations, notably the short rainy season, which promotes the proliferation of larval breeding sites and consequently the abundance of vectors.

The epidemiological data from this study align with the entomological findings. The prevalence of malaria recorded here is comparable to that reported by the National Malaria Control Program in this region (91). Malaria prevalence did not differ significantly between sexes, indicating uniform exposure across genders, as observed in other endemic settings (92, 93). Although the overall sample size and the sub-samples for different age groups were small, our results emphasize an infection risk associated with age. Indeed, children under 10 years of age exhibited a significantly higher prevalence, likely attributable to an immature immune system against *Plasmodium* parasites (6, 93). Besides the ongoing immunization process within this age group, it is also important to note that the same group experiences the highest exposure to mosquito bites. Indeed, when children are tired, they may fall asleep in common places such as the living room or in bedrooms but often do not lower the mosquito net even when it is available. This behavior increases their vulnerability to mosquito bites. The gradual decrease in infection risk with age underscores the acquisition of partial immunity through repeated exposure, which reduces parasitemia and clinical severity while rarely conferring sterilizing immunity (92–94).

Clinically, cases were predominantly mild with no severe malaria observed, consistent with stable moderate transmission zones where semi-immunity mitigates disease progression (95–98). Nevertheless, mild and asymptomatic infections sustain transmission by serving as reservoirs harboring gametocytes capable of infecting mosquitoes (99, 100). These silent infections challenge control programs as they often remain undiagnosed and untreated. The absence of severe malaria likely reflects both acquired immunity and effective local healthcare interventions (101, 102). However, to achieve malaria elimination, sustained efforts including active surveillance targeting these subclinical carriers are essential (103–105).

### 4.1 Study limitation

A major limitation of this study is the relatively small sample size of individuals examined for malaria infection. The research was carried out in Pouth-Kelle, a small and isolated village with an estimated population of approximately 250 inhabitants. Participation in the study was voluntary, which may have introduced selection bias and further limited the representativeness of the sample. Consequently, these factors should be considered when interpreting the findings, and larger studies are taking into account many communities in the Nyong and Kelle Division are recommended to extend these results. Although thin smears were also performed in this study, we should have used molecular tools to characterize the *Plasmodium* species responsible for malaria in this community by screening all collected mosquitoes. Moreover, the collected samples belonging to the *Anopheles gambiae* s.l. complex were not separated during the analyses, although *An. gambiae* was the majority (98%) and *An. coluzzii* represented 2%.

## 5 Conclusion

This study provides a detailed, context-specific analysis of malaria vectors and transmission dynamics in the understudied transitional zone of the Pouth-Kelle community. By identifying vector species, their indoor and outdoor biting behaviors, and seasonal as well as hourly activity patterns, it highlights the complexity of local malaria transmission, including significant outdoor transmission often overlooked by traditional indoor-focused control strategies. The findings emphasize the need for integrated, locally tailored vector control approaches and reinforce the importance of targeting vulnerable groups such as children. This study fills a critical knowledge gap, offering essential data to inform public health policies and optimize malaria control efforts in the Nyong and Kelle Division and similar settings, thereby supporting progress toward national and global malaria elimination goals.

## Data Availability

The raw data supporting the conclusions of this study will be made available by the authors when needed.

## References

[1] Brown JJ Pascual M Wimberly MC Johnson LR Murdock CC. Humidity the overlooked variable in the thermal biology of mosquito-borne disease. Ecol Lett. (2023) 26:1029–49. doi: 10.1111/ele.14228, 37349261 PMC10299817

[2] World Health Organization World malaria report 2024. (2024). Available online at: https://www.who.int/news-room/fact-sheets/detail/malaria (Accessed December 4, 2025).

[3] Medicines for Malaria Venture (MMV). Malaria facts and statistics 2025 (2025). Available online at: https://www.mmv.org/malaria/about-malaria/malaria-facts-statistics-2025 (Accessed December 8, 2025).

[4] Ricci F. Social implications of malaria and their relationships with poverty. Mediterr J Hematol Infect Dis. (2012) 4:e2012048. doi: 10.4084/MJHID.2012.048, 22973492 PMC3435125

[5] Ministère de la Santé Publique (MINSANTE). Guide de prise en charge du paludisme au Cameroun. MINSANTE.2024.pdf. (2024). Available at: https://bibliominsante.cm/server/handle/123456789/1091

[6] World Health Organization. World Malaria Report 2023. Geneva: WHO (2023).

[7] Mbouna AD Tompkins AM Lenouo A Asare EO Yamba EI Tchawoua C. Modelled and observed mean and seasonal relationships between climate, population density and malaria indicators in Cameroon. Malar J. (2019) 18:359. doi: 10.1186/s12936-019-2991-8, 31707994 PMC6842545

[8] PNLP. Plan Strategique nationale de lutte contre le paludisme 2011–2015. Rapport Minsante Cameroun. (2012) 1–82.

[9] Fontenille D Lonchouran C. Malaria epidemiology in Africa: variations in transmission and vector species. Parasitol Today. (1999) 15:173–8.

[10] Wanji S Kamga LM Amvongo-Adjia N Njua-Yafi C Chi H Merki A. Malaria vectors and transmission dynamics in some rural and urban settings in Cameroon. Trans R Soc Trop Med Hyg. (2003) 97:193–9. doi: 10.1016/S0035-9203(03)90008-X

[11] Jetten TH Focks DA Takken W. Environmental and climatic influences on the transmission of vector-borne diseases. Parasitol Today. (1996) 12:114–21.

[12] Castro MC. Malaria transmission and socio-economic factors: a review. Malar J. (2017) 16:163.

[13] Gbaguidi GJ Topanou N Filho WL Begedou K Ketoh GK. Environmental and socio-economic determinants of malaria transmission in West Africa: a systematic review. One Health Outlook. (2025) 7:47. doi: 10.1186/s42522-025-00158-4, 41074119 PMC12512371

[14] Manguin S Garros C Dusfour I Harbach RE Coosemans M. Bionomics, taxonomy, and distribution of *Anopheles* mosquitoes in the context of malaria transmission: a review. Infect Dis Poverty. (2010) 9:83 doi: 10.1186/s40249-020-00739-3

[15] Killeen GF. Characterizing, controlling and eliminating residual malaria transmission. Malar J. (2014) 13:330. doi: 10.1186/1475-2875-13-330, 25149656 PMC4159526

[16] White GB. *Anopheles gambiae* complex and malaria transmission in Africa. Part I. Sources of variation in vectorial capacity. Bull World Health Organ. (1974) 50:439–47.

[17] Ngom EHM Awono-Ambene P Etang J Simard F Antonio-Nkondjio C. *Anopheles* species distribution and malaria transmission dynamics in Sahelian and savannah areas in Cameroon. Malar J. (2014) 13:411. doi: 10.1186/1475-2875-13-411

[18] Lehmann T Hawley WA Grebert H Collins FH. The effective population size of *Anopheles gambiae*. Insect Mol Biol. (2010) 9:357–65. doi: 10.1093/oxfordjournals.molbev.a025925 9501493

[19] Tabue RN Awono-Ambene PH Patchoke S Fongnikin AK Wondji CS Tchouakui M. Distribution of *Anopheles* species in savannah and Sahelian zones of Cameroon. Parasit Vectors. (2017) 10:577.

[20] Sinka ME Rubio-Palis Y Manguin S. The dominant *Anopheles* vectors of human malaria in the Americas: occurrence data, distribution maps and bionomic précis. Parasit Vectors. (2010) 3:72. doi: 10.1186/1756-3305-3-72 PMC2936890 20712879

[21] Antonio-Nkondjio C Ndo C Costantini C Awono-Ambene P Fontenille D. *Anopheles moucheti* and *an. Paludis* are abundant in the forested areas of southern Cameroon and contribute to malaria transmission. Malar J. (2009) 8:50. doi: 10.1186/1475-2875-8-50

[22] Bashar K Tuno N. Seasonal abundance of Anopheles mosquitoes and their association with meteorological factors and malaria incidence in Bangladesh. Parasit Vectors. (2014) 7:442. doi: 10.1186/1756-3305-7-442 PMC4262261 25233890

[23] Bryan JH Hunt RH Adamson R. The ecology and distribution of *Anopheles melas* Theobald in West Africa. Bull Entomol Res. (1987) 77:1–18.

[24] Coetzee M Fontenille D. Anopheline mosquitoes of Africa south of the Sahara. S Afr J Sci. (2004) 98:559–60. doi: 10.1016/j.ibmb.2004.03.012

[25] Corbet PS Service MW. Mosquito Ecology: field Sampling Methods. London: Elsevier Applied Science (1991).

[26] Longo-Pendy N Smith DL Foster WA. Adaptive plasticity of *Anopheles gambiae* complex mosquitoes to diverse ecological conditions. Malar J. (2021) 20:277. doi: 10.1186/s12936-021-03856-5

[27] Akpodiete OJ Otuaro I Eyo JE. *Anopheles stephensi* invasion and ecological adaptation in Africa. Parasit Vectors. (2021) 14:512. doi: 10.1186/s13071-021-04893-y

[28] Rund SSC O’Donnell AJ Gentile JE Reece SE. “Daily rhythms in mosquitoes and their implications for malaria transmission.” Trends Parasitol. (2016) 32:484–496. doi: 10.1016/j.pt.2016.03.005 PMC4931426 27089370

[29] Smith A McKenzie FE Hudson PJ. Variability in biting behavior and host preference of *Anopheles* mosquitoes in Africa. Malar J. (2010) 9:27. doi: 10.1186/1475-2875-9-27

[30] Ndiaye M Gaye A Faye O. Feeding behavior and indoor/outdoor biting preferences of malaria vectors in Senegalese villages. Parasit Vectors. (2018) 11:563. doi: 10.1186/s13071-018-3098-3

[31] Russell TL Govella NJ Azizi S Drakeley CJ Kachur SP Killeen GF. Increased outdoor feeding among residual malaria vector populations following increased use of insecticide-treated nets in rural Tanzania. Malar J. (2011) 10:80. doi: 10.1186/1475-2875-10-80 PMC3084176 21477321

[32] Sherrard-Smith E Skallsjö S Ogutu B. Mosquito feeding behaviour and the importance of human and animal hosts in Kenyan malaria transmission. PLoS One. (2019) 14:e0214941. doi: 10.1371/journal.pone.0214941

[33] Moore SJ Tirados I Flynn M. A behavioural cascade of biting activity in *Anopheles funestus* and *Anopheles arabiensis* to host availability. Nat Commun. (2016) 7:13044. doi: 10.1038/ncomms13044

[34] Killeen GF Govella NJ Okumu FO. Molecular entomology and the ecology of malaria vector control. Adv Parasitol. (2017) 100:275–97.

[35] Smith DL Battle KE Hay SI Barker CM. Characterizing local malaria transmission: scaling vector control to transmission heterogeneity. Adv Parasitol. (2018) 100:61–89. doi: 10.1016/bs.apar.2017.11.003

[36] Tchuinkam T Wondji CS Ndo C. Bionomics of malaria vectors in Cameroonian agro-ecosystems. Parasitol Res. (2012) 110:1689–95.

[37] Ngangue D Toto JC Sandeu MM. Impact of urban agriculture and large-scale development projects on malaria vector diversity and transmission in Cameroon. Malar J. (2020) 19:236.

[38] Kamdem C Fouet C Gamez S. Urbanization fosters adaptive evolution of the malaria vector *Anopheles gambiae*. Proc Natl Acad Sci USA. (2017) 114:2867–72. doi: 10.1073/pnas.1618782114

[39] Mbakop LDN Piameu MLP Ndo C. Malaria vector abundance and transmission related to ecological zones in Cameroon agro-industrial settings. Parasit Vectors. (2019) 12:637.

[40] Mabaso MLH Sharp BL Lengeler C Bruce J. Malaria vector bionomics in poorly studied transitional zones. Infect Dis Poverty. (2019) 8:95. doi: 10.1186/s40249-019-0602-3

[41] Ndo C Awono-Ambene P Mekanda A Antonio-Nkondjio C. Malaria vectors and transmission in forest-savannah transition areas in Cameroon. Malar J. (2015) 14:501.

[42] World Health Organization. Manual on Practical Entomology in malaria. Part II. Methods and Techniques. Geneva: WHO (2013).

[43] Gillies MT de Meillon B. The Anophelinae of Africa South of the Sahara (Ethiopian Zoogeographical Region). Johannesburg: South African Institute for Medical Research (1968).

[44] Gillies MT Coetzee M. A supplement to the Anophelinae of Africa South of the Sahara (Afrotropical Region). South Africa: South African Institute of Medical Research (1987).

[45] Collins FH Mendez MA Rasmussen MO Mehaffey PC Finnerty V Pettigrew LC. A ribosomal RNA gene probe differentiates member species of the *Anopheles gambiae* complex. Am J Trop Med Hyg. (1987) 37:37–41. 10.4269/ajtmh.1987.37.37 2886070

[46] Fanello C Santolamazza F Della Torre A. Simultaneous identification of species and molecular forms of the *Anopheles gambiae* complex by PCR-RFLP. Med Vet Entomol. (2002) 16:461–4. doi: 10.1046/j.1365-2915.2002.00393.x 12510902

[47] Burkot TR Williams JL Schneider I. Identification of plasmodium falciparum-infected mosquitoes by a double antibody enzyme-linked immunosorbent assay. Am J Trop Med Hyg. (1984) 33:783–8. 10.4269/ajtmh.1984.33.783 6385740

[48] Wirtz RA Rooney WF Lee JS Milam DE Rosenberg R. ELISA procedures for detecting *plasmodium falciparum* circumsporozoite antibodies from mosquito salivary glands. Am J Trop Med Hyg. (1987) 36:211–8.

[49] Charan J Biswas T. How to calculate sample size for different study designs in medical research? Indian J Psychol Med. (2013) 35:121–6. doi: 10.4103/0253-7176.116232, 24049221 PMC3775042

[50] World Medical Association. Declaration of Helsinki: ethical principles for medical research involving human subjects. JAMA. (2013) 310:2191–4. doi: 10.1001/jama.2013.281053 24141714

[51] Cheesbrough M. District Laboratory Practices in tropical Countries. Part 2. 2nd ed. Cambridge: Cambridge University Press (2005).

[52] World Health Organization. "Routine examination of blood films for malaria parasites". In: Basic Malaria Microscopy, Learner’s guide. Geneva: WHO (2010). p. 69–76.

[53] World Health Organization. "Parasite density classification". In: Treatment of malaria: Guidelines for malaria, 3rd Edn. Geneva: WHO (2019)

[54] Kiam BC Bouopda-Tuedom AG Mbida Mbida JA Ibrahima I White SJ Tchuenkam PVK . Diversity of anopheline species and malaria transmission dynamics in high-altitude areas of western Cameroon. Malar J. (2025) 24:251. doi: 10.1186/s12936-025-05480-w, 40770357 PMC12330072

[55] Kamdem C . Adaptation of *Anopheles gambiae* complex mosquitoes to urban and peri-urban habitats in Cameroon: a genomic approach. Sci Rep. (2023) 13:10245.

[56] Sinka ME Bangs MJ Manguin S Coetzee M Mbogo CM Hemingway J . The dominant Anopheles vectors of human malaria in Africa, Europe and the Middle East: occurrence data, distribution maps and bionomic précis. Parasit Vectors. (2010) 3:117. doi: 10.1186/1756-3305-3-117, 21129198 PMC3016360

[57] Antonio-Nkondjio C Kerah-Hinzoumbe C Simard F Awono-Ambene P Wondji CS Chouaibou M . Complexity of the malaria vectorial system in Cameroon: contribution of secondary vectors to malaria transmission. J Med Entomol. (2006) 43:1219–25. 10.1603/0022-2585(2006)43[1215:cotmvs]2.0.co;2 17162956

[58] Mombo-Ngoma G Chandelier F Juma O Siba P von Seidlein L. Potential role of *Anopheles paludis* in malaria transmission in changing ecological landscapes. Parasit Vectors. (2016) 9:271.

[59] Lefevre T Gouagna LC Dabire KR Elguero E Fontenille D Renaud F. Species interactions modulate malaria vector competence and transmission dynamics. Proc Biol Sci. (2017) 284:20161978.

[60] Smith DL Battle KE Hay SI Barker CM Scott TW McKenzie FE. Ross, Macdonald, and a theory for the dynamics and control of mosquito-transmitted pathogens. PLoS Pathog. (2012) 8:e1002588. doi: 10.1371/journal.ppat.1002588 PMC3320609 22496640

[61] World Health Organization. Guidelines for Integrated Vector Management. Geneva: WHO (2012).

[62] Killeen GF Smith TA McKenzie FE Mbogo CM Knols BG Beier JC. Preventing childhood malaria in Africa by protecting adults from mosquitoes with insecticide-treated nets. PLoS Med. (2007) 4:e229. doi: 10.1371/journal.pmed.0040229 PMC1904465 17608562

[63] Kahamba NF Mmbando BP Kweka EJ. Geospatial modelling of dry season habitats of the malaria vectors in East Africa highlights seasonal breeding site dynamics. Malar J. (2024) 23:64. Available at: https://parasitesandvectors.biomedcentral.com/articles/10.1186/s13071-024-06119-6

[64] Masse RS Vythilingam I Fornace K Othman H Liu X Jaafar AJ . Impact of environmental factors on the bionomics of Anopheles mosquito vectors of zoonotic malaria: A narrative review. One Health. (2025) 21:101141. doi: 10.1016/j.onehlt.2025.101141 PMC12301798 40727445

[65] Whittaker C Lines J Ghana S. Seasonal variation in *Anopheles* mosquito populations and implications for malaria control. Parasit Vectors. (2023) 16:98. doi: 10.1186/s13071-023-05678-4

[66] Coetzee M Koekemoer LL. Molecular systematics and insecticide resistance in the Anopheles funestus group: a review. Acta Trop. (2013) 128:399–406. doi: 10.1016/j.actatropica.2013.01.004, 23317045

[67] Tene Fossog B Ayala D Lo E Nkahe D Costantini C. Temporal and spatial dynamics of malaria vectors in equatorial forest regions. Malar J. (2015) 14:104. doi: 10.1186/s12936-015-0644-0

[68] Killeen GF McKenzie FE Foy BD Bogh C Beier JC. The *Anopheles gambiae* complex: vector behavior and implications for malaria control. Am J Trop Med Hyg. (2006) 74:609–16.

[69] Dambach P Gueye AS Diallo M Tine RC Sauerborn R Becker N. Feeding behaviors of *Anopheles nili* in Senegal and implications for malaria transmission. Parasit Vectors. (2018) 11:313.

[70] Nzioki M Onyango F Odera J. Early evening outdoor biting and malaria risk in Kenya. J Med Entomol. (2023) 60:450–60. doi: 10.1093/jme/tjac118

[71] Braack L Macdonald MB. Outdoor biting by malaria vectors: what are the options for intervention? Malar J. (2025) 25:18. doi: 10.1186/s12936-025-05689-9 PMC12784499 41350710

[72] Durnez L Coosemans M. Residual transmission of malaria: an old issue for new approaches. Insects. (2013) 4:126–42.

[73] Tondossama A Moussa M Kamdem C. Indoor biting rates of *Anopheles gambiae* s.l. and malaria transmission in Central Africa. Malar J. (2023) 22:108.

[74] Hayes CC Schal C. Review on the impacts of indoor vector control on domiciliary pests: good intentions challenged by harsh realities. Proc Biol Sci. (2024) 291:20240609. doi: 10.1098/rspb.2024.0609 PMC11265923 39043243

[75] Fornadel CM Norris LC Glass GE. Characterizing *Anopheles* vector population dynamics and behavior in eastern Africa. J Med Entomol. (2011) 48:25–37.

[76] Edwards HM Norris LC Hamieda AS Watz N. Biting behavior and habitat utilization of *Anopheles* species in forested ecotopes. Parasit Vectors. (2019) 12:345.

[77] Mbogo CM Snow RW Kabiru EW Ouma JH Githure JI Beier JC. Low-level malaria transmission and implications for vector control in Kenya. Am J Trop Med Hyg. (1993) 49:547–53. 10.4269/ajtmh.1993.49.245 8357087

[78] Keïta Y Diarra M Griffin JT. Outdoor malaria transmission and vector behavior in West Africa. Malar J. (2021) 20:276. doi: 10.1186/s12936-021-03800-4

[79] Carnevale P Manguin B. Indoor and outdoor malaria transmission dynamics in endemic African regions. Parasitol Today. (2021) 37:795–803. doi: 10.1016/j.pt.2021.06.005

[80] Otolorin GR Castellanos ME Adegboye OA McBryde ES. Integrated vector management for malaria control: a review of approaches and effectiveness. Trans R Soc Trop Med Hyg. (2025) 119:1223–1232. doi: 10.1093/trstmh/traf084 PMC12580886 40847646

[81] Govella NJ Ferguson HM Matovu E. Entomological surveillance of outdoor exposure to malaria vectors. Trends Parasitol. (2013) 29:419–20. doi: 10.1016/j.pt.2013.06.002

[82] Fillinger U Govella NJ Ferguson HM. Innovative tools for outdoor malaria vector control. Annu Rev Entomol. (2023) 68:145–67.

[83] Killeen GF Seyoum A Knols BGJ. Rationalizing historical successes of malaria control in Africa in terms of mosquito resource availability management. Malar J. (2014) 13:19. doi: 10.1186/1475-2875-13-19 15331823

[84] Harris C White GB Killeen GF. Effects of large-scale distributions of insecticide-treated nets in rural Tanzania on *Anopheles gambiae* blood feeding behavior and entomological inoculation rates. Malar J. (2006) 5:77. doi: 10.1186/1475-2875-5-77

[85] Cohuet A Simard F Wondji CS Antonio-Nkondjio C Awono-Ambene P Fontenille D. High malaria transmission intensity due to Anopheles funestus in a village of Savannah-forest transition area in Cameroon. J Med Entomol. (2004) 41:901–5. doi: 10.1603/0022-2585-41.5.901, 15535619

[86] Ossè RA Yaro AS Zoh MG. Involvement of *Anopheles nili* in *plasmodium falciparum* transmission in southern Benin. Malar J. (2019) 18:388. doi: 10.1186/s12936-019-2792-0 PMC6489317 31036025

[87] Baldwin AL Smith JH Clark RM. The impact of sexual behavior on exposure to infectious diseases: a review. J Epidemiol. (2012) 22:345–56. doi: 10.2188/jea.JE20110124

[88] Djoufounna J Djiappi-Tchamen J Mayi MP Foyet JV Ndjeunia-Mbiakop P Joko S . Malaria transmission dynamics in Makenene, a forest–savanna transition zone, in Centre region, Cameroon. IJID One Health. (2026) 11:100113. doi: 10.1016/j.ijidoh.2026.100113

[89] Elanga-Ndille E Binyang A Ndo C. Entomological indicators of malaria transmission and insecticide resistance profile of *Anopheles gambiae* at the early phase of irrigated rice farming in the forest area of Central Cameroon [version 1; peer review: 1 approved with reservations, 1 not approved]. Wellcome Open Res. (2020) 5:190. doi: 10.12688/wellcomeopenres.16064.1

[90] Aduvukha GR Abdel-Rahman EM Mutanga O Odindi J Tonnang HEZ. Spatial-temporal coupling of malaria vector habitat suitability and biting probability. Spat Spatiotemporal Epidemiol. (2026) 56:100777. doi: 10.1016/j.sste.2025.100777 41714056

[91] National Institute of Statistics (NIS): Low reduction in malaria parasite prevalence; in children under 5 in Cameroon by 2023; let’s prevent malaria in central, southern and eastern Cameroon. (2023). p. 5.

[92] Centers for Disease Control and Prevention. Malaria – transmission and risk factors. (2024). Available online at: https://www.cdc.gov/malaria/about/biology/transmission.html

[93] Smith T Ross A Maire N Tediosi F. Relationships between immunity and exposure: implications for malaria epidemiology. Nat Rev Immunol. (2006) 6:616–28. doi: 10.1038/nri1880

[94] Snow RW Guerra CA Noor AM Myint HY Hay SI. The global distribution of clinical episodes of plasmodium falciparum malaria. Nature. (1997) 434:214–7. 10.1038/nature03342 PMC3128492 15759000

[95] Doolan DL Dobaño C Baird JK. Acquired immunity to malaria. Clin Microbiol Rev. (2009) 22:13–36. doi: 10.1128/CMR.00025-08, 19136431 PMC2620631

[96] Marsh K Kinyanjui S. Immune effector mechanisms in malaria. Parasite Immunol. (2006) 28:51–60. doi: 10.1111/j.1365-3024.2006.00808.x, 16438676

[97] Langhorne J Ndungu FM Sponaas AM Marsh K. Immunity to malaria: more questions than answers. Nat Rev Immunol. (2019) 9:725–32. 10.1038/ni.f.205 18563083

[98] Langhorne J Buffet P Galinski MR Good MF Harty JT. Immunity to malaria: more questions than answers. Nat Immunol. (2008) 9:725–32. doi: 10.1038/ni.f.205, 18563083

[99] Bousema T Drakeley C. Epidemiology and infectivity of plasmodium falciparum and plasmodium vivax gametocytes in relation to malaria control and elimination. Clin Microbiol Rev. (2011) 24:377–410. doi: 10.1128/CMR.00051-10, 21482730 PMC3122489

[100] Ferreira RAM da Silva-Nunes M Sampaio V d S. The role of asymptomatic infections in malaria transmission. Malar J. (2017) 16:221. doi: 10.1186/s12936-017-1840-x

[101] Bousema T Okell L Felger I Drakeley C. Asymptomatic malaria infections: detectability, transmissibility and public health relevance. Nat Rev Microbiol. (2014) 12:833–40. doi: 10.1038/nrmicro3364, 25329408

[102] Lindblade KA Steinhardt L Samuels A Kachur SP Slutsker L. The silent threat: asymptomatic parasitemia and malaria transmission. Expert Rev Anti-Infect Ther. (2013) 11:623–39. doi: 10.1586/eri.13.45, 23750733

[103] White NJ Pukrittayakamee S Hien TT Faiz MA Mokuolu OA. Malaria. Lancet. (2014) 383:723–35. doi: 10.1016/S0140-6736(13)60024-0, 23953767

[104] Idro R Marsh K John CC Newton CRJC. Cerebral malaria: mechanisms of brain injury and strategies for improved neurocognitive outcome. Pediatr Res. (2005) 57:60–8. doi: 10.1203/01.PDR.0000145990.59304.50 PMC3056312 20606600

[105] Stresman G Greenhouse B Miller J Shinn A Malar B. Targeting asymptomatic reservoirs of malaria infections to achieve elimination. Curr Opin Infect Dis. (2014) 27:481–8. doi: 10.1097/QCO.0000000000000105

